# The Effect of Viral Infection on the Growth of HoneySweet GM Plum Trees

**DOI:** 10.3390/plants15060903

**Published:** 2026-03-14

**Authors:** Petr Komínek, Marcela Komínková, Jana Brožová

**Affiliations:** Ecology, Diagnostics and Genetic Resources of Agriculturally Important Viruses, Fungi and Phytoplasmas, Czech Agrifood Research Center, Drnovská 507, Ruzyně, 161 00 Prague, Czech Republic; marcela.kominkova@carc.cz (M.K.); jana.brozova@carc.cz (J.B.)

**Keywords:** plum pox virus, HoneySweet, transgenic plum, field trial, tree growth, mixed viral infection

## Abstract

Plum pox virus (PPV) is one of the most destructive pathogens affecting stone fruit trees. It causes sharka disease and severe yield losses. The genetically modified plum cultivar ‘HoneySweet’ was developed to provide long-lasting resistance to PPV via RNA interference. Long-term field trials of ‘HoneySweet’ have been conducted in the Czech Republic since 2001, involving the artificial inoculation of the cultivar with PPV alone, and with apple chlorotic leaf spot virus (ACLSV) and prune dwarf virus (PDV) in combination. This study evaluates the impact of viral infection on tree growth after 24 years in the field. Growth parameters—trunk cross-sectional area (TCSA) and canopy volume—were measured and analysed using ANOVA and Tukey’s test. The results show that infected trees exhibit significantly reduced growth compared to non-infected controls, with the strongest inhibition observed in trees inoculated with PPV + PDV + ACLSV. The presence of ACLSV had the most pronounced negative effect on growth, while PDV did not significantly influence tree vigour. These findings emphasise the importance of using virus-free rootstocks and certified planting material to prevent growth suppression in HoneySweet orchards.

## 1. Introduction

Plum pox virus (PPV), which belongs to the species *Potyvirus* *plumpoxii*, genus *Potyvirus* and the family *Potyviridae*, is one of the most harmful viruses affecting stone fruits, where it causes a disease called sharka or plum pox [1]. The basic methods for reducing the harmfulness of this virus in fruit trees include the use of certified virus-free planting material and the use of varieties with a sufficient degree of resistance to prevent damage to the fruit and thus yield reduction. In the case of plums, the search for resistant varieties is more difficult because there is no known variety that is fully resistant to the virus. Plum varieties that are tolerant to infection by this virus are used, as they do not cause damage to the fruit. Trees of virus-tolerant varieties can thus be infected by aphids in field conditions, and the virus is still present in high concentrations in the tissues of these tolerant varieties and can spread uncontrollably by aphids to other susceptible plants in the vicinity [2]. Another option is to use plums with a hypersensitive reaction to PPV infection, where the spread of the virus in the plant is stopped after the initial infection. A typical example is the Jojo variety [3]. In order to create a plum tree that is truly resistant to the virus, a genetically modified (GM) plum tree carrying the CP gene of PPV was developed by an international scientific consortium. The aim of this genetic modification is to induce resistance to PPV. This GM plum tree was designated as clone C5, later as the HoneySweet variety. The HoneySweet plum tree was subsequently tested in greenhouse and field conditions for resistance to PPV [4,5,6]. The mechanism of action, i.e., resistance to PPV, is determined by the IRSH (inverted repeat split by hairpin) construct, which consists of two inverted copies of the PPV CP gene separated by a hairpin loop, controlled by a pair of CaMV35S promoters. This construct induces a strong RNAi mechanism, which leads to the formation of siRNA and epigenetic methylation of target sequences [7].

In field conditions, one of the longest-running trials with HoneySweet plums was conducted in Romania, where HoneySweet’s resistance to PPV was verified and its environmental safety documented [8,9].

Other field trials with HoneySweet cultivation have been conducted in Poland and Spain [10].

However, the longest-running field trial is in the Czech Republic, where this GM plum tree has been grown in an experimental orchard in Prague since 2001, with the trial scheduled to end in 2027. The aim of the trial is to research the resistance of this plum tree after artificial infection with the primary target of transgenosis, which is the plum pox virus. The field trial also includes mixed infections with two other common plum viruses to study the interactions of all the viruses used with the GM HoneySweet plum trees. These viruses are the apple chlorotic leafspot virus (ACLSV) of the species *Trichovirus mali*, genus *Trichovirus*, family *Betaflexiviridae*, and the prune dwarf virus (PDV) of the species *Ilarvirus PDV*, genus *Ilarvirus*, family *Bromoviridae*.

The results of research into the resistance of HoneySweet plum trees in a field trial in Prague have already been published, confirming that GM HoneySweet plum trees are highly resistant to PPV infection and that heterologous infection with ACLSV or PDV does not suppress resistance to PPV [11].

The fertility of GM plums in this field trial was not evaluated because the trial must comply with the Czech Republic’s law (Act No. 78/2004 Coll., on genetically modified organisms), which requires the prevention of transgene spread into the surrounding environment. For this reason, all fruits on GM plum trees in the field trial must be harvested before ripening so that seeds from these fruits cannot enter the environment, germinate and give rise to an uncontrolled population of genetically modified plants. The field trial is therefore located in an area isolated from related cross-pollinating plants, in this case at Prague International Airport, where this small experimental orchard is surrounded by extensive grassy areas without shrubs or trees. Three Jojo plum trees are also growing in the orchard. These were used for environmental protection research, i.e., to verify the possibility of pollen transfer from GM HoneySweet plums to non-transgenic vegetation. The presence of GM embryos in Jojo plums has not been proven, so there has been no effective transfer of GM pollen to non-transgenic vegetation [12].

This study focuses on another aspect of research into PPV-resistant plums, specifically the evaluation of the long-term (24 years) impact of infection by one or more viruses on the growth and vitality of these plums.

## 2. Results

**Figure 1:**
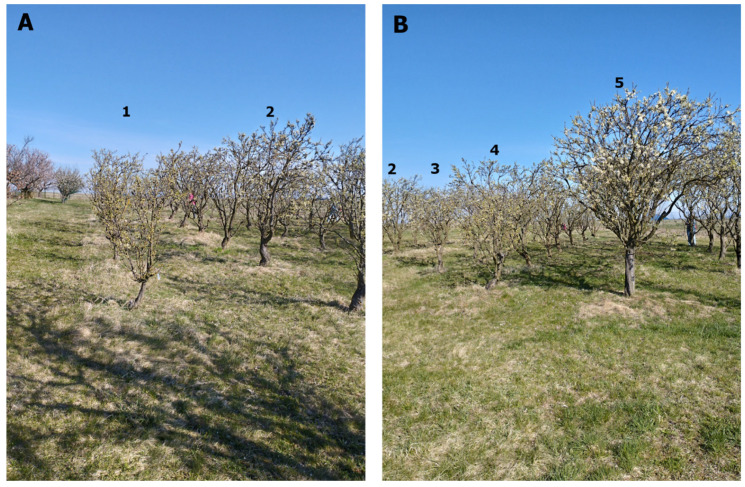
Differences in growth between variants of the field trial with GM HoneySweet plums. The photographs were taken at the beginning of the trial rows and are aimed in the direction of the rows formed by the trial variants. The variant number is always indicated above the relevant row. See Table 1 for variant identification. The photographic record of the differences between variants is divided into two images to clearly show the differences in growth between the individual rows. (**A**) Shot of variants 1 and 2. (**B**) Photo taken at variant 5, documenting the difference in growth compared to variants 2–4.

The variants of the experiment infected with various combinations of viruses generally showed lower tree growth than the uninfected control. The most significant suppression of HoneySweet plum growth was caused by infection with three viruses, i.e., PPV- + PDV + ACLSV. Other variants of infection, i.e., PPV + ACLSV, PPV + PDV, and PPV alone, also caused severe growth suppression compared to the uninfected control. See Figure 1, where the differences in growth between the individual variants are clearly visible. In the measured parameters, this fact was most evident in the volume of tree canopies. Trees infected with PPV + PDV + ACLSV had a canopy volume 7.8 times smaller than that of the uninfected control. Trees infected with PPV + PDV had a canopy volume 2.5 times smaller, trees infected with PPV + ACLSV had a canopy volume four times smaller, and trees infected with PPV had a canopy volume 2.7 times smaller than that of the uninfected control. With regard to TCSA, both variants containing ACLSV (PPV + PDV + ACLSV and PPV + ACLSV) achieved significantly lower values than the uninfected control. The PPV + PDV and PPV variants had TCSA values close to those of the uninfected control.

The results of the canopy volume and TCSA measurements are summarised in Table 1. All measured data are available in the Supplementary Materials.

The analysis of variance showed significant differences between the variants in both parameters. Canopy volume F = 62.83, *p* = 2.86 × 10^−18^. Trunk cross-sectional area F = 7.21, *p* = 1.30 × 10^−4^.

**Figure 2:**
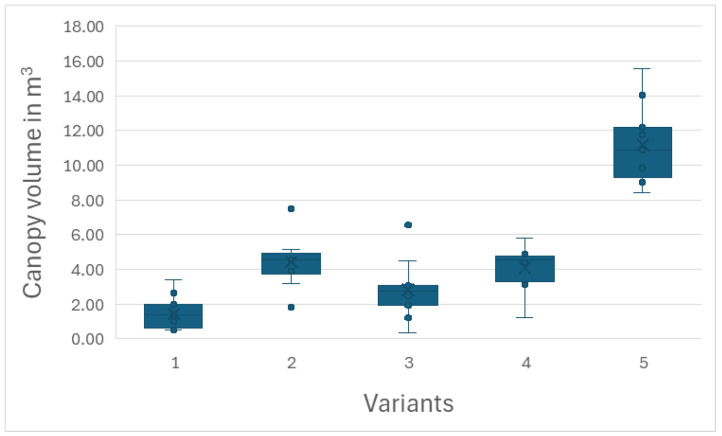
Box plot showing differences between the variants of the experiment—canopy volume parameter.

Variant 1 (PPV + PDV + ACLSV) has the lowest value in the canopy volume parameter. Compared to variant 1, variant 3 (PPV + ACSLV) achieved twice the average canopy volume, but the difference is not significant. Another significantly larger canopy volume was measured for variants 2 (PPV + PDV) and 4 (PPV), which differ significantly (*p* < 0.05) from variant 1. The uninfected control (variant 5) again has a significantly larger canopy volume and achieved a significant difference (*p* < 0.001) in this parameter compared to all virus-infected variants. See also Figure 2. Detailed results are available as Supplementary Materials.

**Figure 3:**
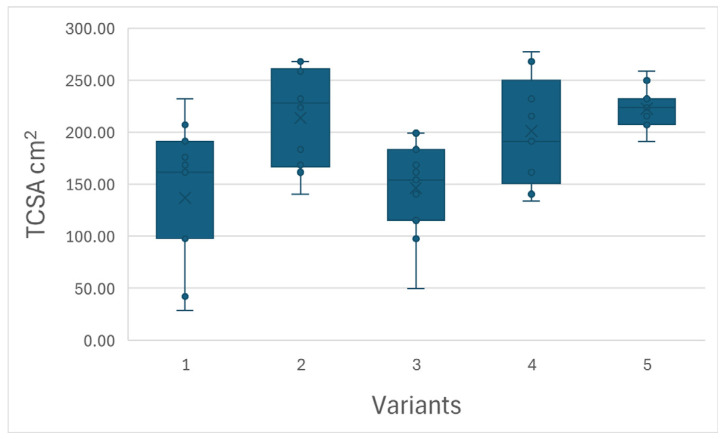
Box plot showing the differences between the variants of the experiment—trunk cross-section area parameter.

In the TCSA parameter, the two variants with the lowest values form a group—variant 1 (PPV + PDV + ACLSV) and variant 3 (PPV + ACLSV). The remaining three variants form a group whose members do not differ significantly from each other (*p* > 0.05): variants 2 (PPV + PDV), 4 (PPV) and 5 (uninfected control). Variant 1 differs from all members of the group consisting of variants 2, 4 and 5. Variant 3 differs from variants 2 and 5. See also Figure 3.

### HoneySweet Fruitfulness in the Experiment

The fruitfulness of HoneySweet trees was not evaluated. On the one hand, it was not possible to let the fruit ripen for legislative reasons, and on the other hand, fruit set was minimal throughout the experiment, mainly due to the absence of suitable trees as pollinators. Although three Jojo plum trees grow in close proximity to the trial, this was not sufficient to pollinate more than 50 HoneySweet trees. In addition, under the conditions of our field trial, the Jojo variety blooms later than HoneySweet, and there may not be sufficient pollen transfer to HoneySweet. Nor did pollen transfer appear to occur in the opposite direction, i.e., from HoneySweet to the later-flowering Jojo. In our published work, we described that we did not detect any transgene transfer to Jojo [12].

## 3. Discussion

The primary objective of the field experiment was to evaluate the resistance of the HoneySweet GM plum tree to viral infection. During the experiment, it was found that infection with viruses and combinations of viruses affects the growth of the experimental trees compared to uninfected trees. In this paper, we evaluate this finding.

Many factors influence the growth, vitality and fertility of fruit trees after planting in a permanent location. These include factors related to the plant itself—the choice of fruit species, variety and rootstock; environmental factors—soil, water regime and climatic conditions; biotic factors—diseases and pests; and agrotechnical measures—nutrition, irrigation, pruning and pest control. The most common way of regulating the growth of fruit trees in fruit growing practice is to use a suitable rootstock, although the exact mechanism of the rootstock’s effect on growth is not yet fully understood [13].

In the case of the HoneySweet GM plum field experiment, its homogeneous establishment eliminated the influence of rootstock, variety, year, location and agrotechnical measures on tree growth. HoneySweet trees were infected by grafting as one-year-old plants. The inoculation grafts were left to grow in order to achieve sustained infection pressure.

The data clearly show that persistent PPV infection pressure, either alone or in combination with other viruses, reduces the growth of HoneySweet trees. All trees in the artificially infected variants had a significantly lower canopy volume than the control uninfected trees. The growth suppression is permanent; even after 24 years of growth, the infected trees did not reach the height of the control, uninfected trees.

There are clear differences in canopy volume between the individual variants with virus combinations. The lowest growth was observed when using three viruses. The second lowest growth was observed when using PPV together with ACLSV. A significantly larger tree canopy volume was achieved when using PPV together with PDV. This variant achieved the same canopy volume as using PPV alone, so it can be concluded that PDV infection did not have a significant effect on tree growth. The strongest inhibitory effect on canopy volume is therefore the use of ACLSV in a mixture of inoculated viruses.

In terms of trunk cross-section, the variants were divided into two groups, with the lower trunk cross-section including the PPV + PDV + ACLSV and PPV + ACLSV variants. The remaining three variants, i.e., PPV + PDV, PPV alone and the uninfected control, did not differ significantly from each other. Once again, we can conclude that ACLSV in combination with other viruses has a strong negative influence on the growth of the experimental trees.

It is also important to mention the significance of the parameters we monitored, i.e., canopy volume and trunk cross-sectional area, for assessing tree fertility. According to a study [14] conducted on Santa Rosa plums in India, both parameters had the same effect on fruit yield. However, in most cases, both parameters are used without comparison [15]. TCSA is considered a more reliable parameter because it is measured in the lower part of the tree, where the conditions for canopy formation are created [16]. In addition, canopy volume as an indicator can be influenced by the training of the tree canopy and the intensity of pruning [17].

To determine the effect of ACLSV and PDV alone on HoneySweet, it would have been appropriate to include these variants in the experiment from the beginning. Since the experiment focused on researching resistance to PPV, variants without PPV were not necessary and were therefore not included in the experiment from the beginning. Although these variants were later added to the field experiment, due to the time delay, they can no longer be compared with the original variants in terms of growth assessment.

Our experiment with the HoneySweet GM plum is unique not only in its duration (24 years), but also in its design, which includes simple and mixed infections with multiple viruses. Another unique feature is the simultaneous evaluation of both resistance and the effect of viruses on tree growth and vitality. Not many studies have been published on the effect of multiple viral infections on the growth, fertility, or resistance of fruit trees. Peach stunt disease is an example of the synergistic effect of mixed infection with Prunus necrotic ringspot virus (PNRSV) and PDV in peach trees [18]. Mixed infection with these two viruses has also been described in cherries, where it caused reduced vegetative growth of trees compared to single infection with these viruses [19]. In sour cherries, the effect of PNRSV and PNRSV + cherry virus A infection on the content of active compounds in fruits has been described [20]. For plums, there are publications focusing on multiplex virus diagnostics, which provide a list of detected viruses, their percentage occurrence and the occurrence of various combinations of viruses [21,22,23].

The results of the experiment involving artificial inoculation of HoneySweet GM plums with various viruses and combinations thereof provide recommendations for the potential commercial production planting of this plum variety. The results show a significantly negative effect of viral infection on the growth and vitality of HoneySweet plums, especially in the case of PPV and ACLSV viruses. Permanent infection pressure, induced in experimental conditions by grafting infectious inoculum, could occur in commercial planting through the use of infected rootstock. Therefore, it is necessary to emphasise the need to use certified virus-free rootstocks for HoneySweet plums, as is the case with any other viral pathogens in conventional, i.e., unmodified fruit trees. The certification of rootstock and variety planting material, therefore, remains the basic method of controlling viral diseases in permanent agricultural crops such as fruit trees. The advantage of using resistant varieties, including HoneySweet, which we have researched, is their permanent resistance to natural viral infection pressure.

## 4. Materials and Methods

### 4.1. Experimental Trees

**Figure 4:**
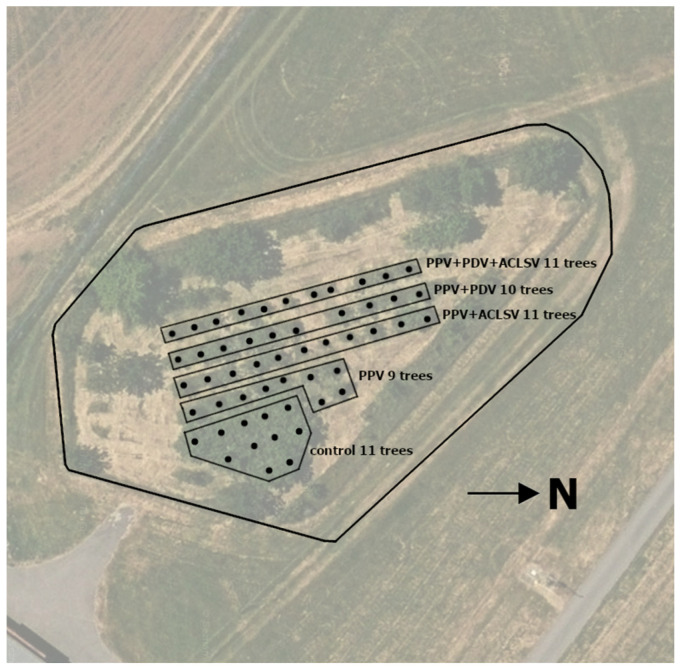
Map of the HoneySweet plum field experiment based on orthophotography.

The field experiment with HoneySweet plum trees includes a total of four variants with different combinations of viruses and an uninfected control. See Table 1 for an overview of the experiment variants. See Figure 4 for the experiment layout.

All trees in the experiment were prepared under the same conditions and at the same time. HoneySweet plum grafts were obtained from Dr. Scorza of the USDA (Kearneysville, WV, USA). The plum tree was then grafted onto prepared St. Julien rootstocks in a screenhouse in Prague-Ruzyně. One-year-old HoneySweet plants were inoculated with viruses according to the experiment variants using grafting. PPV, PDV, and ACLSV viruses were used in the experiment because infections with these viruses occur in plums in Europe. According to a survey conducted in Czechia [24], PPV is the most widespread virus in plum trees (74% of tested plants), followed by PNRSV (27%), PDV (2%), ApMV (1%) and ACLSV (1%). Similarly, in Romania [25], the most widespread virus in plum orchards is PPV (32%), followed by PNRSV (8.1%) and PDV (1.2%).

The viral inoculum came from a virus collection maintained at our institution (Czech Agrifood Research Center Prague, formerly Research Institute of Crop Production Prague). Inoculation grafts were taken from trees infected only with the desired virus. The presence of the desired virus and the absence of other potential viruses in the source trees were verified using ELISA and RT-qPCR [11].

The infected plants were planted in the field trial site at Prague-Ruzyně International Airport. The inoculum was left to grow in order to create permanent infection pressure. In the following years, the trees were evaluated for resistance on leaves and flowers. All agronomic measures such as pruning, nutrition and pesticide protection were applied equally to all experimental plants. After canopy formation, the trees were not pruned further and were left to grow freely, so the overall growth of the trees and the volume of their canopies were influenced by only one variable, namely the presence and quality of the viral inoculum (one, two or three viruses).

The presence of viruses in HoneySweet plums and growing inocula was verified by analysing leaves and flowers using ELISA and RT-qPCR annually throughout the duration of the field trial [11]. Similarly, symptoms on the leaves of HoneySweet plums and growing inocula (Figure S1) were evaluated each year.

In 2025, an assessment of the growth of individual trees (the plants were 24 years old at the time) was carried out, which is the subject of this work.

### 4.2. Surroundings of the Experiment

In addition to HoneySweet plum trees, the experimental orchard also includes a number of Karola (a variety susceptible to PPV) and Harlayne (a variety resistant to PPV) apricot trees, originally planted around the entire perimeter of the orchard. The apricots were originally infected with various strains of PPV for resistance research and as a source of infectious inoculum for GM plums. During the experiment, the Karola apricots were removed because the infectious pressure was maintained by inoculum growing directly on the infected HoneySweet plums.

A number of Jojo plum trees, also infected with various strains of PPV for resistance research, were originally planted in the experimental orchard. During the course of the experiment, only three Jojo trees uninfected with PPV remained. They were used to investigate the possibility of transgenic pollen transfer to surrounding vegetation.

Beyond the boundaries of the experimental orchard are the grassy areas of Prague International Airport, with no shrubs or trees that could be a source of viral inoculum or to which transgenic pollen could be transferred.

### 4.3. Evaluation of HoneySweet Plum Growth

#### 4.3.1. Trunk Cross-Sectional Area

For fruit trees, trunk cross-sectional area (TCSA) is often used to evaluate growth, as used, for example, in the case of apples (*Malus* × *domestica*) by [26] or for plums (*Prunus domestica*) by [14]. We therefore performed manual measurements of the trunk circumference at a height of 20 cm above the ground and calculated the trunk cross-sectional area (TCSA, cm^2^) according to the formula

$$A=\frac{C^{2}}{4\pi }$$

where

*A*—area (of the trunk cross-section)
*C*—trunk circumference

#### 4.3.2. Canopy Volume

We chose canopy volume as another parameter for evaluating tree growth due to the visually striking differences between the experimental variants.

Manual measurements were used to determine the volume of the tree canopy. A tape measure was used for the measurements, which were taken by two people. For each plant, the height of the entire plant, the height of the trunk, and the width and thickness of the canopy were measured. One measurement was taken for each parameter. For simplicity, the canopy of the experimental trees was compared to a simple upside-down cone, where the apex of the cone lies at the point where the primary branches of the canopy branch out and the base of the cone is the upper plane of the canopy. To calculate the base of the cone, it was necessary to determine its radius, which was calculated from the measured values of the width and thickness of the canopy [27]. The volume of the canopy was then calculated as the volume of the cone according to the formula

$$V=\frac{1}{3}*\pi *r^{2}*h$$

where

*V*—volume of the cone (tree canopy)
*r*—radius of the cone base
*h*—height of the cone

### 4.4. Analysis of the Obtained Data

The effect of individual experiment variants on the measured tree growth parameters was evaluated using one-way analysis of variance followed by Tukey’s test using Microsoft^®^ Excel^®^ for Microsoft 365 MSO (version 2601, Microsoft Corporation, Redmond, WA, USA). Before conducting the one-way ANOVA, the normality was assessed using the Shapiro–Wilk test, applied separately within each variant. Homogeneity of variances was evaluated using Levene’s test, performed independently for canopy volume and trunk cross-sectional area. In both cases, the assumption of homogeneity was not violated (*p* > 0.05), which justified the use of Tukey’s HSD for post hoc comparisons.

## Figures and Tables

**Table 1:** Evaluation of the growth of HoneySweet GM plums infected with viruses and their combinations.

Variant Number	Number of Trees	Inoculated Viruses	Canopy Volume m^3^	Trunk Cross-Sectional Area cm^2^
1	11	PPV + PDV + ACLSV	1.44 ± 0.93 c	136.83 ± 67.06 b
2	10	PPV + PDV	4.42 ± 1.45 b	213.59 ± 46.84 a
3	11	PPV + ACLSV	2.82 ± 1.63 b	146.23 ± 45.24 b
4	9	PPV	4.07 ± 1.32 b	201.14 ± 51.71 a
5	11	uninfected control	11.16 ± 2.19 a	222.52 ± 19.96 a

Note: Values represent the mean of the variant, the standard deviation and the different lowercase letters indicate statistically significant differences between the evaluated variants (*p* < 0.05).

## Data Availability

All data supporting the findings of this study are available within the article and Supplementary Materials.

## Supplementary Materials

The following supporting information can be downloaded at: https://www.mdpi.com/article/10.3390/plants15060903/s1, Figure S1: Symptoms of PPV on the leaves of a growing inoculum shoot (center of image) and a GM plum shoot with no symptoms on the leaves (right).; Table S1: Measured data—complete; Table S2: Measured data—summary; Table S3: ANOVA—canopy volume; Table S4: ANOVA—TCSA; Table S5: Tukey’s test—canopy volume; Table S6: Tukey’s test—TCSA; Table S7: ANOVA—graphs.

## Abbreviations

The following abbreviations are used in this manuscript:

CP	coat protein
RNA	ribonucleic acid
CaMV	cauliflower mosaic virus
USDA	United States Department of Agriculture
