# Supplementary material for: The Effect of Viral Infection on the Growth of HoneySweet GM Plum Trees
Source: Plants (Basel). 2026 Mar 14;15(6):903. doi: 10.3390/plants15060903 (PMC13030480; doi:10.3390/plants15060903)
Supplement: Supplementary file 1 [file plants-15-00903-s001.zip › Figure S1 with caption.pdf]

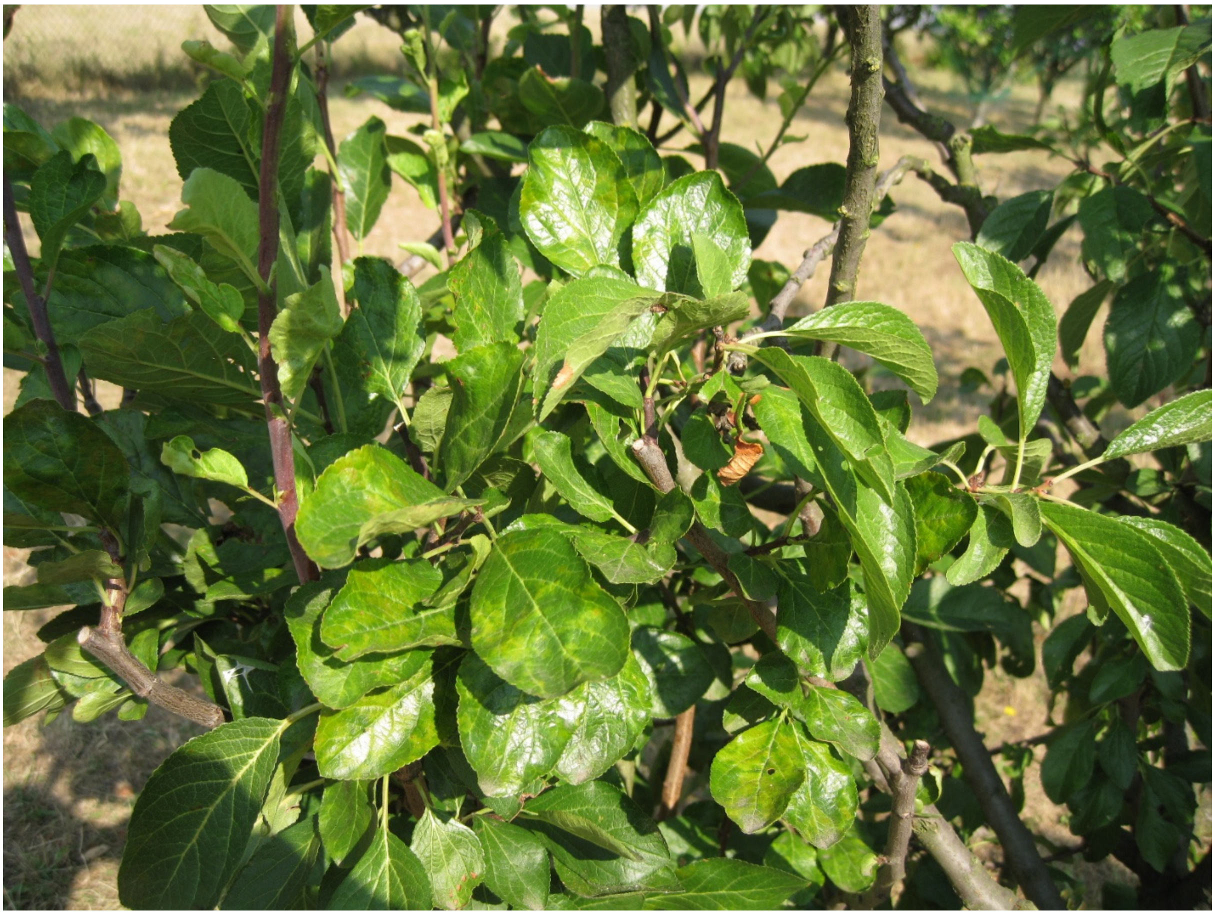

Figure S1: Symptoms of PPV on the leaves of a growing inoculum shoot (center of image) and a GM plum shoot with no symptoms on the leaves (right).
